# The Other-Race and Other-Species Effect during a Sex Categorization Task: An Eye Tracker Study

**DOI:** 10.3390/bs10010024

**Published:** 2020-01-01

**Authors:** Merve Bulut, Burak Erdeniz

**Affiliations:** Department of Psychology, Izmir University of Economics, İzmir 35330, Turkey; burak.erdeniz@ieu.edu.tr

**Keywords:** other-race effect, other-species effect, sex categorization, eye tracking, face processing, holistic processing, face inversion effect

## Abstract

Sex categorization from faces is a crucial ability for humans and non-human primates for various social and cognitive processes. In the current study, we performed two eye tracking experiments to examine the gaze behavior of participants during a sex categorization task in which participants categorize face pictures from their own-race (Caucasian), other-race (Asian) and other-species (chimpanzee). In experiment 1, we presented the faces in an upright position to 16 participants, and found a strong other-race and other-species effect. In experiment 2, the same faces were shown to 24 naïve participants in an upside-down (inverted) position, which showed that, although the other-species effect was intact, other-race effect disappeared. Moreover, eye-tracking analysis revealed that in the upright position, the eye region was the first and most widely viewed area for all face categories. However, during upside-down viewing, participants’ attention directed more towards the eye region of the own-race and own-species faces, whereas the nose received more attention in other-race and other-species faces. Overall results suggest that other-race faces were processed less holistically compared to own-race faces and this could affect both participants’ behavioral performance and gaze behavior during sex categorization. Finally, gaze data suggests that the gaze of participants shifts from the eye to the nose region with decreased racial and species-based familiarity.

## 1. Introduction

Sex categorization from faces is an important skill for social interaction and mate selection, a crucial ability developed throughout evolution [1]. Humans and many non-human primates have developed a sexually dimorphic face, which helps them to use facial cues when identifying the sex of others [2]. Previous studies showed that humans can successfully categorize the sex of other people’s faces in a very short duration (26 to 75 ms) [3,4]. These studies suggest that compared to recognition [5], sex categorization from faces is a very rapid process (approximately 150 ms) [6], even when the faces were blurred with different filters [7].

Face processing for own-race faces was shown to be more efficient compared to other-race faces. This phenomenon is defined as the other-race effect in the literature [8]. Similarly, humans are more successful at processing own-species faces compared to other-species faces, a phenomenon referred to as the other-species effect [9]. Even though there are several studies examining the other-race and other-species effect during face recognition, there is a lack of understanding of sex categorization of faces from other-race and other-species, and controversial findings were observed [3,10]. For example, O’Toole and colleagues [3] showed the other-race effect with Caucasian and Japanese faces in a sex categorization task, whereas Zhao and Bentin [10] did not find such an effect in their study with Chinese and Israeli participants. Furthermore, a thorough search of the relevant literature yielded only one study that directly tested sex categorization in other-species faces and suggested that humans can successfully categorize the sex of macaque monkeys based on their faces without training [11,12]. These studies indicate that humans might be able to categorize the sex of other species from their faces, yet there remains a gap in the literature on how they could achieve this. Therefore, the current study aimed to address the question of how human adults can successfully perform a sex categorization task with other-race and other-species faces.

Examining the gaze behavior of participants while face processing helps us to understand the underlying mechanism of face perception. Previous studies consistently point out that the eye is the most informative and diagnostic area during a sex categorization task with own-race faces [13,14], but several eye-tracking studies indicate that an observer’s gaze initially shifts to the nose region [15,16] rather than the eyes. Several studies also indicate that during face perception tasks, the gaze pattern of participants can change for other-race and other-species faces. For example, one study [17] found that during race categorization, participants mostly attend to the eye region of own-race faces, but to the nose and mouth region of other-race faces. These studies indicate different gaze behavior of participants for other-race and other-species faces compared to own-race faces. However, to the best of our knowledge, none of these previous studies examined the gaze behavior of participants during a sex categorization task with other-race and other-species faces. Therefore, in the current study, we aimed to examine the gaze behavior of participants during a sex categorization task with own-race, other-race, and other-species faces.

In experiment 1, we aimed to investigate whether humans can differentiate the sex of other-race and other-species faces, and if so, which facial cues they utilize for successful categorization. In order to achieve this, we presented Caucasian (own-race), Asian (other-race), and Chimpanzee (other-species) faces to a group of Caucasian participants and asked them to indicate whether the face is male or female. Following that, in experiment 2 we examined the effect of holistic processing, defined as processing different facial regions as a whole [18]. Several studies indicate that own-race faces are processed more holistically compared to other-race and other-species faces (for a review, see reference [19]). In order to test this theory, in experiment 2 we used the same facial stimuli from experiment 1, but presented them in an upside-down position. Furthermore, we recorded the gaze behavior of participants during both experiments in order to reveal how the facial integration occurs for own-race, other-race, and other-species faces during sex categorization. We hypothesized that in experiment 1, participants will show more accurate categorization performance in own-race and own-species faces (compared to other-race and other-species faces respectively), but for experiment 2, own-race and own-species advantage will disappear due to the loss of holistic processing strategy.

## 2. Experiment 1

### 2.1. Materials and Methods

#### 2.1.1. Participants

In Experiment 1, 26 Turkish students from Izmir University of Economics volunteered to participate in the study in exchange for course credits. After the calibration processes ended, we had 16 participants (8 females) (*M*_age_ = 22.31, *SD*_age_ = 3.59). A power analysis was performed by using Power ANalysis for GEneral Anova Designs (v0.2) (Austin TX, USA) software with a medium effect size (*d* = 0.45). This analysis showed that a power value of 0.83 is adequate for the current experimental design with 16 participants. None of the participants had any neurological or psychiatric disorder, or were under the effect of any drugs. All participants were right-handed [20] and had normal vision. All participants signed a written informed consent form and were informed about the study. Furthermore, none of the participants reported being in an East Asian country for more than six months, or any familiarity with East Asian culture or people. Participants using eyeglasses or contact lenses were excluded from the study. Finally, in order to avoid calibration problems, we asked participants not to wear any eye makeup on the day of the experiment. The study was conducted in accordance with the Declaration of Helsinki and approved by the Izmir University of Economics ethics committee.

#### 2.1.2. Stimuli

Frontal looking photographs of Caucasian (14 males, 14 females), East-Asian (14 males, 14 females), and chimpanzee (four males, four females) faces with a neutral expression were selected for own-race, other-race face, and other-species categories, respectively. All Caucasian faces with neutral expressions were selected from Radbaud University, Radbaud Face Database (http://www.socsci.ru.nl:8180/RaFD2/RaFD [21]. For the other-race category, neutral expression, frontal pose photographs of East-Asian (Chinese) University students were selected from CUHK Face Sketch Database (CUFS) (http://mmlab.ie.cuhk.edu.hk/archive/facesketch.html) [22]. Finally, frontal looking neutral chimpanzee face photographs were selected from the James & Other Apes book [23]. All of the chimpanzees were over three years old, the critical period for a sexually dimorphic face [24,25]. All chimpanzee face photographs were taken in a controlled way (see http://jamesmollison.com/books/james-other-apes/).

#### 2.1.3. Apparatus

Firstly, all face stimuli were cropped from their natural facial outline and placed on a black background with a grayscale (454 × 340 pixels). SMI (Senso Motoric Instruments) Experiment Center version 3.4 was used to present faces to participants via a desktop computer (3 GHz) on a 22-inch monitor. In order to allow participants to lead their first fixation themselves, faces appeared in one of four equally divided quadrants of the screen at random. Caucasian, Asian and chimpanzee faces were presented as separate blocks. The order of the blocks was counterbalanced between participants and there was a within-block randomization of the stimuli for each participant. Faces stayed on the screen for 5 s in an upright position. Participants responded by using the N and M keys (counterbalanced between participants) on the keyboard in order to indicate the sex of the face in the photograph.

#### 2.1.4. Eye Tracking

While participants were performing the sex categorization task, their eye movements were tracked at a sampling rate of 250 Hz with the Remote Eye-Tracking Device (RED 250, SMI). The iView X system (SMI) was used to record gaze data. The experimental chamber was soundproofed and the experimental session was observed by a camera from another room. Participants were seated with their eyes 60–65 cm away from the monitor, which is a typical distance for human interaction [26]. The eye-tracking device was set just below the monitor. In order to stabilize participants’ heads, a chin rest was used. A calibration process was applied with a black background, and nine red-point fixation procedures were performed for each participant. 

The SMI Experiment Center AOI (Area of Interest) Editor was also used to perform area of interest analysis. Areas of interest were drawn based on previous research, which showed that the eyes, nose, mouth, cheeks, and jaw are critical regions for sex categorization from faces [15,16,27]. Based on previous studies, the AOI for eyes were defined to include the eyebrows. Furthermore, we excluded the upper part of the nose, because previous research showed that this particular regions is crucial for holistic processing [16,28] and is not sexually dimorphic [15].

#### 2.1.5. Procedure

Before the experiment, participants were first informed about the study, and signed an informed consent form. Demographic information, including the participant’s age, sex, dominant hand, medical history (neurological and/or psychiatric disorder), and vision problems were collected. Before starting the experiment, participants were instructed that, by using their right index finger, they were expected to categorize the faces on the computer screen as either female or male (using the keyboards M and N keys). The time limit for categorization of the faces was set to 5 s, after which time the next stimuli appeared. In order to practice, all participants completed a short version of the experiment with 10 faces that were not included in the main experiment. After the practice session, the participant’s head was positioned according to the eye-tracking device, and stabilized with a chin rest. Participants were instructed not to move their heads during the experiment. After calibration and validation processes were successfully completed, the main experiment started.

Before the presentation of each face, a fixation cross was presented at the center of the screen. A 500 ms fixation trigger was arranged for each fixation cross, such that the next stimuli did not appear until the participant had fixated for 500 ms. There was a rest period of 30 s between each block, during which participants could close their eyes or look outside the screen without moving their head. All faces were presented only once during the experiment. At the end of the experiment, participants received a debriefing about the purpose of the study.

### 2.2. Results

#### 2.2.1. Behavioral Results

In the first experiment, the percentage of correct sex categorization was calculated for each face category. Results revealed that Caucasian (*M* = 95.17, *SE* = 1.07, 95% CI [93.07, 97.27]), Asian (*M* = 89.96, *SE* = 1.31, 95% CI [87.39, 92.53]) and chimpanzee faces (*M* = 64.84, *SE* = 3.65, 95% CI [57.69, 71.99]) were successfully categorized based on their sex at a significantly higher than chance level (50%); *t* (15) = 42.35, *p* < 0.001, *r* = 0.99, *t* (15) = 30.43, *p* < 0.001, *r* = 0.98; *t* (15) = 4.07, *p* < 0.01, *r* = 0.72, respectively. Furthermore, a one-way repeated measures ANOVA showed that accuracy was significantly affected by the category of faces, *F* (1.45, 21.70) = 140.11, *p* < 0.001, *η_p_*^2^ = 0.57. Planned contrasts revealed that there was a significant other-race (comparing Caucasian vs. Asian), *F* (1, 15) = 11.39, *p* < 0.01, *r* = 0.66) and other-species effect (comparing Caucasian and Asian vs. chimpanzee; *F* (1, 15) = 171.59, *p* < 0.001, *r* = 0.96). These results showed that participants were more accurate in determining the sex of the faces from their own race and own-species faces compared to other-race and other-species.

#### 2.2.2. Gaze Behavior

During gaze behavior analyses, only correct trials were included [15], and fixations on non-face regions were excluded from the analyses. Successful fixations were defined as maintaining the gaze for at least 100 ms [29,30]. A preliminary analysis showed that neither gender of the observer nor sex of the face had main or interaction effects for any of the measurements (behavioral and gaze data) (all *p* values > 0.05). Therefore, these two independent variables concatenated for all analyses.

**Figure 1:**
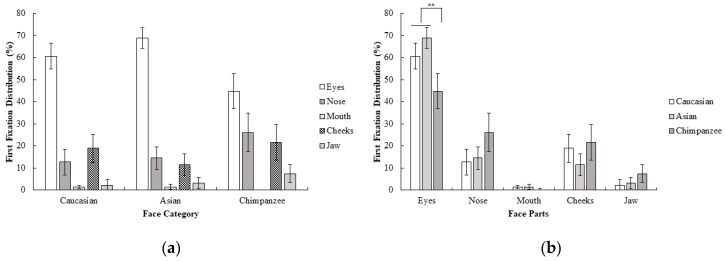
Mean percentage (with 95% CI) of first fixation frequency as a function of (**a**) face category and (**b**) face part in Experiment 1.

The percentage of first fixation frequency of participants were subjected to a 3 (face category; Caucasian, Asian, chimpanzee) x 5 (face part; eyes, nose, mouth, cheeks, jaw) repeated measure ANOVA. Results show that the interaction between the face category and face region was statistically significant; *F* (3.08, 46.21) = 4.56, *p* < 0.01, *η_p_*^2^ = 0.23. Additionally, contrast analysis revealed that eyes were more frequently fixated on as the first area than the other face parts for Caucasian and Asian faces compared to chimpanzee faces; *F* (1, 15) = 9.18, *p* < 0.01, *r* = 0.62. None of the other contrasts reached the statistical significance threshold, all *F* values < 4.15 and all *p* values > 0.05. Furthermore, none of the contrasts between Caucasian and Asian faces on the first fixations reached a statistical significance threshold, all *F* values < 3.03, all *p* values > 0.05 (Figure 1). Results suggests that Caucasians mostly focus on the eyes first in all face categories when categorizing the sex of the face. However, the difference between eyes and other face parts were more dramatic in human (Caucasian and Asian) faces, and there was a more highly distributed pattern of first fixations across the eyes, nose, and cheeks in chimpanzee faces.

**Figure 2:**
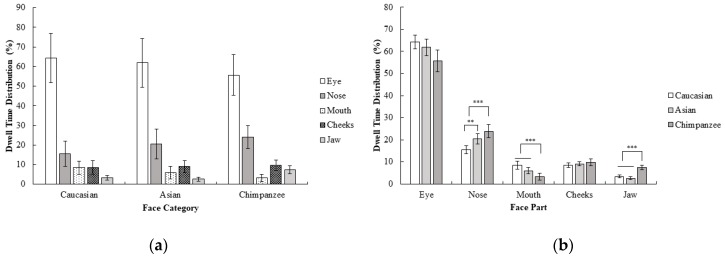
Mean percentage (with 95% CI) of dwell time as a function of (**a**) face category and (**b**) face part in Experiment 1.

Furthermore dwell time distribution for each face category and region was calculated by correcting the AOI size, since dwell time measurement can be highly affected by the AOI size (dwell time on each face part were divided into its AOI size) and a repeated measure ANOVA of 3 (face category; Caucasian, Asian, chimpanzee) × 5 (face part; eyes, nose, mouth, cheeks, jaw) was performed. Results revealed a significant interaction between the face category and face part, *F* (2.16, 32.39) = 4.81, *p* < 0.05, *η_p_*^2^ = 0.24. According to these results, the nose was more attended to than the mouth, cheeks, and jaw in chimpanzee faces compared to in human faces; *F* (1, 15) = 7.14, *p* < 0.05, *r* = 0.57. On the other hand, the mouth was more attended to than cheeks and jaw in human faces compared to chimpanzee faces; *F* (1, 15) = 27.49, *p* < 0.001, *r* = 0.80. Other contrasts did not reach the significance level, all *F* values < 4.25, and all *p* values > 0.05. Furthermore, the nose was more highly focused on compared to other areas in Asian faces than in Caucasian faces, *F* (1, 15) = 11.89, *p* < 0.01, *r* = 0.66. Other contrasts did not reach the significance level, all *F* values < 4.21, all *p* values > 0.05 (Figure 2). Corresponding with first fixations, the eyes were the area that received the most attention in all face categories. In general, there was a similar pattern in dwell time distribution across different face categories, except that the nose was focused on for a longer period of time in chimpanzee and Asian faces compared to Caucasian faces, and the mouth was focused on for a longer in human compared to chimpanzee faces even though there was no difference between these comparisons in the first fixation of participants.

## 3. Experiment 2

### 3.1. Materials and Methods

#### 3.1.1. Participants

For the second experiment, 26 naïve right-handed students who have normal vision from Izmir University of Economics volunteered to enroll in exchange for course credits. After the calibration processes, 24 participants (12 females) (*M*_age_ = 22.08, *SD*_age_ = 2.30) remained. A power analysis was performed by using Power ANalysis for GEneral Anova Designs (v0.2) software with a medium effect size (*d* = 0.45). This analysis showed that a power value of 0.95 is adequate for the current experimental design with 16 participants. All participants signed an informed consent form and were debriefed after the experimental session. The inclusion and exclusion criteria were the same as for the first experiment, such that participants with a neurological or psychiatric disorder or under the effect of any drug were excluded. Furthermore, none of the participants reported being in an East Asian country more than six months or any familiarity with East Asian culture or people.

#### 3.1.2. Procedure

In experiment 2, the equipment, stimuli, and procedure were the same as in the first experiment, except that all stimuli were presented upside-down (rotated 180°). This allowed us to study the holistic processing effect on faces in the sex categorization task [31].

### 3.2. Results

#### 3.2.1. Behavioral Results

Similar to in the first experiment, the percentage of accuracy for each face category was calculated for the second experiment. Results revealed that Caucasian (*M* = 81.99, *SE* = 1.62, 95% CI [78.81, 85.17]) and Asian faces (*M* = 77.59, *SE* = 2.09, 95% CI [73.49, 81.69]) were successfully categorized based on their sex at a significantly higher than chance level; *t* (23) = 19.80, *p* < 0.001, *r* = 0.68; *t* (23) = 13.20, *p* < 0.001, *r* = 0.60, respectively. However, participants failed to categorize the sex of the chimpanzee faces (*M* = 55.21, *SE* = 3.76, 95% CI [47.84, 62.58]); *t* (23) = 1.39, *p* > 0.05. A one-way repeated measures ANOVA on percentage of accuracy revealed a significant main effect of face categories; *F* (1.32, 31.40) = 29.85, *p* < 0.001, *η_p_*^2^ = 0.57. After rotation, the other-species effect was still present (comparing Caucasian and Asian versus chimpanzee; *F* (1, 23) = 51.03, *p* < 0.001, *r* = 0.83), but the other-race effect disappeared (comparing Caucasian versus Asian; *F* (1, 23) = 3.81, *p* > 0.05) indicating that there was no difference in participants’ categorization accuracy between Caucasian and Asian faces.

#### 3.2.2. Gaze Behavior

**Figure 3:**
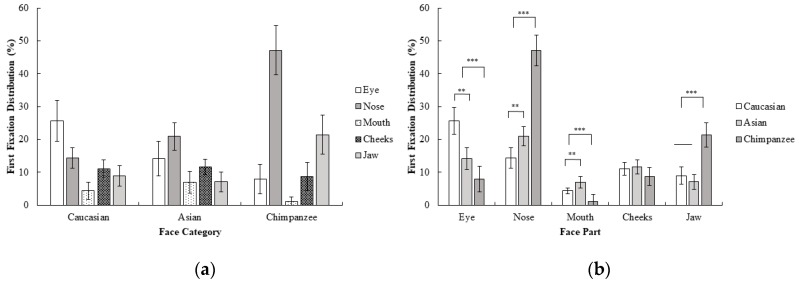
Mean percentage (with 95% CI) of first fixation frequency (%) as a function of (**a**) face category and (**b**) face part in Experiment 2.

A 3 (face category; Caucasian, Asian, chimpanzee) × 5 (face part; eyes, nose, mouth, cheeks, jaw) repeated measures ANOVA for the first fixation revealed a significant interaction effect between the face category and face part; *F* (4.84, 111.28) = 26.62, *p* < 0.001, *η_p_*^2^ = 0.54. Planned contrast analysis revealed that the eye was more frequently first fixated upon compared to other areas in human faces than in chimpanzee faces; *F* (1, 23) = 35.54, *p* < 0.001, *r* = 0.78. However, for chimpanzee faces, the nose was more frequently first fixated on than the mouth, cheeks, and jaw, compared to human faces; *F* (1, 23) = 44.62, *p* < 0.001, *r* = 0.81. The mouth on the other hand, was more frequently first fixated on than the cheeks and jaw in human faces compared to chimpanzee faces; *F* (1, 23) = 21.00, *p* < 0.001, *r* = 0.69. Finally, the jaw was more frequently first fixated on than the cheeks in chimpanzee faces compared to the human faces; *F* (1, 23) = 15.21 *p* < 0.01, *r* = 0.63. Contrasts between Caucasian and Asian faces revealed a significant advantage of the eye over other areas in Caucasian compared to Asian faces for first fixation frequency; *F* (1, 23) = 14.71, *p* < 0.01, *r* = 0.62. On the other hand, there was a significant advantage for the nose over the mouth, cheeks, and jaw in Asian faces compared to Caucasian faces *F* (1, 23) = 8.64, *p* < 0.01, *r* = 0.52. Furthermore, in Asian faces, the mouth was more frequently first fixated on than the cheeks and jaw, compared to Caucasian faces, *F* (1, 23) = 9.15, *p* < 0.01, *r* = 0.53. No statistically significant difference in first fixations were observed for the cheeks and jaw between the Caucasian and Asian faces, *F* (1, 23) = 1.01, *p* > 0.05 (Figure 3). When these results are compared with the first experiment, first fixations on Caucasian faces were generally on eyes as in Experiment 1, although a more distributed pattern across different face parts was observed in Experiment 2. In Asian faces, the difference between the eye and nose area in the first experiment disappeared and participants did not show any significant tendency between the eyes and nose for their first fixations in Experiment 2. Furthermore, participants changed their first fixation from the eye (Experiment 1) to the nose area in Experiment 2, which might suggest that a chimpanzee face might be perceived as a non-face object in the first fixations as a result of the inversion.

**Figure 4:**
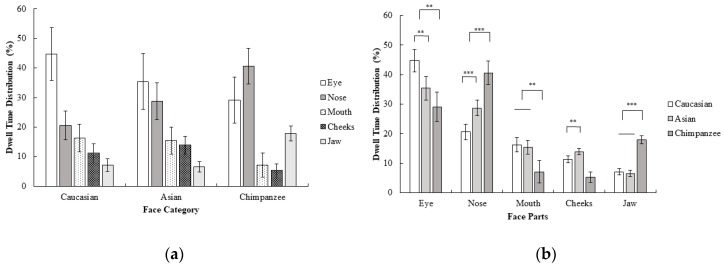
Mean percentage (with 95% CI) of dwell time (%) as a function of (**a**) face category and (**b**) face part in Experiment 2.

A 3 (face category; Caucasian, Asian, chimpanzee) × 5 (face part; eyes, nose, mouth, cheeks, jaw) repeated measures ANOVA on dwell time revealed a significant interaction effect of the face category and face part; *F* (3.31, 76.15) = 15.73, *p* < 0.001, *η_p_^2^* = 0.41. Planned contrasts revealed that the eyes were focused on more than other areas in humans compared to chimpanzee faces; *F* (1, 23) = 8.34, *p* < 0.01, *r* = 0.52. The nose, on the other hand, was more focused on than the mouth, cheeks, and jaw in chimpanzee faces compared to human faces; *F* (1, 23) = 25.70, *p* < 0.001, *r* = 0.73. The mouth was more focused on than the cheeks and jaw in human faces compared to in chimpanzee faces; *F* (1, 23) = 10.83, *p* < 0.01, *r* = 0.57. The jaw, on the other hand, was more focused on than the cheeks in chimpanzee faces compared to in human faces, *F* (1, 23) = 85.33, *p* < 0.001, *r* = 0.89. Furthermore, the eye was more focused on than other areas in Caucasian compared to Asian faces; *F* (1, 23) = 9.26, *p* < 0.01, *r* = 0.54. The nose on the other hand, was more focused on than other areas in Asian faces compared to in Caucasian faces; *F* (1, 23) = 22.10, *p* < 0.001, *r* = 0.70. Finally, the cheeks were more focused on than the jaw in Asian faces compared to Caucasian faces; *F* (1, 23) = 13.70, *p* < 0.01, *r* = 0.61 (Figure 4). In general, dwell time measurements were consistent with first fixations. Compared with experiment 1, the dwell time pattern of participants in Caucasian faces was similar in Experiment 2, although the time spent focusing on the eye area decreased. Furthermore, the nose was more focused on in Asian and chimpanzee faces as dwell time in the eye decreased (compared to Experiment 1) as a result of inversion.

## 4. Discussion

The current study investigated the other-race and other-species effect during a sex categorization task. The behavioral results of the first experiment showed that humans can successfully categorize chimpanzee faces at a significantly higher than chance level, and there was a strong other-race and other-species effect for the upright faces. However, the results of the second experiment showed that the other-race effect disappeared when the faces were rotated, indicating that participants were equally successful in categorizing the sex of Caucasian and Asian faces, although the categorization accuracy decreased for all face categories in general. Based on the behavioral results of two experiments, the disappearance of the other-race effect in Experiment 2 can be evaluated as important evidence indicating a greater tendency to use a holistic processing strategy for own-race compared to other-race faces. Similarly, Hancock and Rhodes [32] reported that the inversion effect was more disruptive for own-race faces compared to other-race faces in a face recognition task with Caucasian and Asian faces [33,34]. When faces were presented upside-down, the behavioral performance of own-race faces was affected more negatively than other-race faces. Relevant literature suggests that humans might process their own-race faces more holistically compared to other-race faces and this might be the reason for the underlying mechanism of the other-race effect [19]. In the current study, there was a strong other-race effect when faces were presented in upright position, but this disappeared with inversion. We believe that, consistent with the literature, this is evidence that humans process their own-race faces more holistically compared to other-race faces. One possible explanation why the other-species effect was still present after the inversion might be that the unfamiliarity of chimpanzee faces compared to Asian faces. In fact, in experiment 2, other-species faces might be perceived as non-face objects, since participants could not even successfully categorize the sex of these faces.

The gaze behavior of participants in the first experiment revealed that in upright position, the eyes were the first and the most fixated upon area. The eyes were followed by the nose in dwell time measurements, although the nose did not differ from the cheeks and jaw for first fixation frequency. However, Sæther and colleagues [15] found no difference between eyes, nose, or cheeks on first fixation frequency, and found the nose to be the most focused on area in a sex categorization task with own-race faces. They furthermore revealed that the most focused on area within the nose was the infraorbital margin (the lower side of the eye socket adjacent to the nose). One possible explanation for the differences between our study and Sæther and colleagues [15] might due to the exclusion of this area in our definition of the nose area of interest, because it is not sexually dimorphic [15]. We furthermore revealed that participants show a similar gaze pattern for the other-race and other-species faces in an upright position.

The results of Experiment 2 showed that the eye region was more highly focused on in human faces compared to chimpanzee faces when faces were presented upside-down. In addition, the eye area of the Caucasian faces was more focused on compared to eye region of the Asian faces in terms of both dwell time and first fixation frequency. The nose, on the other hand, was more focused on for other-race and other-species faces. These findings are the first of their kind in the literature, and suggest that inverting the faces influences the configural processing of facial features during a sex categorization task. 

In conclusion, the current study showed that humans have a similar gaze pattern for own-race, other-race, and other-species faces in an upright position (with the eye being the first and most highly focused on area) during sex categorization. On the other hand, their gaze pattern shifted more towards the eye region with increased racial and species-based familiarity, and shifted more towards the nose region in other-race and other-species faces in response to upside-down presentation of the faces. These results suggest that holistic processing of faces is highly influenced by the race and species of the facial stimuli, and its spatial orientation during presentation. Furthermore, the current study provides evidence for a strong holistic processing strategy for the own-race compared to other-race faces based on the behavioral results. Future studies should consider these factors while investigating face perception and sex categorization in humans. Finally, conducting several other studies with different races and species would be necessary to extend the discussion of other-race and other-species effects.
